# DTI and MR Volumetry of Hippocampus-PC/PCC Circuit: In Search of Early Micro- and Macrostructural Signs of Alzheimers's Disease

**DOI:** 10.1155/2012/517876

**Published:** 2011-06-12

**Authors:** F. Palesi, P. Vitali, P. Chiarati, G. Castellazzi, E. Caverzasi, A. Pichiecchio, E. Colli-Tibaldi, F. D'Amore, I. D'Errico, E. Sinforiani, S. Bastianello

**Affiliations:** ^1^Neuroradiology Unit, IRCCS Foundation National Neurological Institute C. Mondino, 27100 Pavia, Italy; ^2^Department of Physics “A. Volta”, University of Pavia, 27100 Pavia, Italy; ^3^Department of Computer Engineering and Systems Science, University of Pavia, 27100 Pavia, Italy; ^4^Neurology Unit, IRCCS Foundation National Neurological Institute C. Mondino, 27100 Pavia, Italy; ^5^Department of Radiology, University of Pavia, 27100 Pavia, Italy

## Abstract

Hippocampal damage, by DTI or MR volumetry, and PET hypoperfusion of precuneus/posterior cingulate cortex (PC/PCC) were proposed as biomarkers of conversion from preclinical (MCI) to clinical stage of Alzheimer's disease (AD). This study evaluated structural damage, by DTI and MR volumetry, of hippocampi and tracts connecting hippocampus to PC/PCC (hipp-PC/PCC) in 10 AD, 10 MCI, and 18 healthy controls (CTRL). Normalized volumes, mean diffusivity (MD), and fractional anisotropy (FA) were obtained for grey matter (GM), white matter (WM), hippocampi, PC/PCC, and hipp-PC/PCC tracts. In hippocampi and hipp-PC/PCC tracts, decreased volumes and increased MD were found in AD versus CTRL (*P* < .001). The same results with lower significance (*P* < .05) were found in MCI versus CTRL. Verbal memory correlated (*P* < .05) in AD with left hippocampal and hipp-PC/PCC tract MD, and in MCI with FA of total WM. Both DTI and MR volumetry of hippocampi and hipp-PC/PCC tracts detect early signs of AD in MCI patients.

## 1. Background

Preclinical detection of Alzheimer's disease (AD) is important to start an early therapeutic treatment, and it will be even more crucial in the next few years, as soon as new drugs will be available. Mild cognitive impairment (MCI) is often the preclinical stage of AD. However, some patients with MCI revert to normal cognitive status, while others, with slow disease progression, remain in this prodromic stage without presenting dementia in their life [1]. 

To detect which patients with MCI will convert in AD in the immediate future, an in vivo biomarker is not currently available. CSF tau, phospho-tau, and amyloid measurements are in development [2, 3] but require lumbar puncture; therefore, a noninvasive imaging marker is more appealing for screening outpatients without hospital admission. With this purpose, volumetric MRI measures of mesiotemporal atrophy demonstrated to have some prognostic value [4, 5]. Compared to these volumetric measures of macrostructural damage, diffusion tensor imaging (DTI) indexes of microstructural damage within mesiotemporal lobe have shown to better discriminate MCI from controls [6, 7] and to better detect MCI converters [8–10]. 

DTI is sensitive to both grey and white matter subtle abnormalities. While in AD degeneration mainly affects grey matter [11], recent evidences also found an early white matter involvement [12, 13]. It is a matter of debate whether degeneration directly affects the myelin, but a secondary wallerian degeneration certainly drives disconnection of associative cortical areas from the medial temporal lobe. In MCI, the earliest affected area by disconnection seems to be the precuneus/posterior cingulate cortex (PC/PCC), consistently found hypoperfused by many PET studies [14] and related to the conversion in AD [15]. A recent study combining PET and volumetric MRI showed that PC/PCC hypoperfusion follows medial temporal atrophy through posterior cingulum white matter degeneration [16]. Furthermore, several studies using resting state fMRI showed a functional disconnection between hippocampus and PC/PCC [17, 18]. 

In this study, to identify sensitive in vivo biomarkers of risk for conversion from MCI to AD, the microstructural damage was measured by DTI in the hippocampus and in the white matter between hippocampus and PC/PCC. The DTI indexes of microstructural damage were compared to volumetric indexes of macrostructural damage in MCI and AD patients, using as control a group of healthy elderly volunteers.

## 2. Methods

### 2.1. Subjects

Twenty patients were consecutively recruited through the Memory Clinic of the Neurological Institute C. Mondino, Pavia, Italy, among patients suffering from subjective or objective memory complaint.

Exclusion criteria were age > 80, a history of overt depression [19] or other psychiatric diseases, significant cerebrovascular disease [20], and lack of any daily living activity. In this small patients group, exclusion of subjects over 80 years minimizes the contribution of confounding variables of age and of age-related diseases, particularly cerebrovascular disease.

Neuropsychology examination by a standardised battery evaluated different cognitive domain [21]. In this study, we only considered the global measure of cognitive impairment as expressed by MMSE [22], and verbal memory as expressed by short story recall [21].

After clinical and neuropsychological examinations, 10 patients were diagnosed with amnestic mild cognitive impairment [1] and 10 with mild probable Alzheimer's disease (NINCDS2-ARDA criteria [23]) (Table 1).

Twenty elderly subjects were recruited on a volunteer base through a local recreational association (“Argento Vivo,” i.e., “Live silver,” Bereguardo, PV). After clinical and neuropsychological examinations, two volunteers affected by vascular cognitive impairment were excluded. The remaining 18 healthy volunteers were included as control group (CTRL) and underwent the MRI session. 

### 2.2. MRI Acquisition

All data were acquired on a 1.5 Tesla MRI scanner (Intera, Philips Gyroscan, Koninklijke, The Netherlands) using an eight-channel head (SENSE) third-party coil.

All subjects were scanned with a structural MRI protocol, including a dual turbo spin echo (TSE) sequence (proton density and T2), a volumetric T1-weighted sequence, and diffusion tensor imaging (DTI) data. Functional MRI data during resting state are not considered in this study. Additional FLAIR images were acquired only in patients with some punctuate lesions on PD-T2 images. While most patients and volunteers showed some punctuate lesions, only few showed confluent lesions, in any case never involving more than a third of total white matter. Although subtle cerebrovascular disease may be a factor concurring to cognitive impairment in MCI and AD, for the purpose of this study the total lesion load was not assessed.

Diffusion tensor imaging (DTI) data were acquired using a single-shot EPI spin echo sequence (TR/TE = 11800/70 ms) with a *b*-value of 900 s/mm^2^, applying diffusion gradients along 15 directions. Sixty axial slices with no slice gap were acquired (FOV = 224 mm, acquisition matrix = 88 × 90, reconstruction matrix = 96 × 96, 2.5 mm isotropic voxel, number of averages = 3). 

Volumetric T1-weighted data was collected using a fast field echo sequence (TR/TE = 8.6/4 ms, flip angle 8°) and one hundred seventy sagittal slices with a thickness of 1.2 mm (FOV = 240 mm, matrix = 192 × 192, in-plane resolution 1.25 × 1.25 mm, reconstruction matrix = 256 × 256).

### 2.3. MR Imaging Analysis

**Figure 1:**
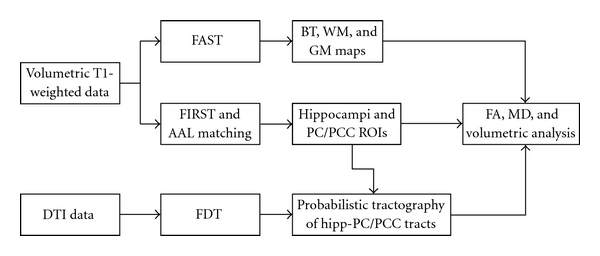
Study design schematic. DTI data were preprocessed by FDT (FSL) to obtain MD and FA maps. Structural T1 data were corrected in intensity by FreeSurfer and segmented in brain, grey and white matter volumes by FAST (FSL). Hippocampi and PC/PCC were segmented automatically by FIRST and AAL template registration and then registered on DTI maps. Tracts connecting hippocampus to PC/PCC (hipp-PC/PCC) were identified by probabilistic tractography within FDT and then registered back on structural T1 data. Normalized volumes, MD, and FA values were obtained for GM, WM, hippocampi, PC/PCC, and hipp-PC/PCC tracts.

Image analysis was performed on a workstation with Linux Ubuntu 9.10, running SPM8 (Wellcome Department of Cognitive Neurology, http://www.fil.ion.ucl.ac.uk/) on MATLAB 7.9 (The MathWorks, Natick, Mass, USA http://www.mathworks.com/), FreeSurfer (http://surfer.nmr.mgh.harvard.edu/), and FSL (FMRIB Software Library, http://www.fmrib.ox.ac.uk/fsl/) software (Figure 1). 

A nonparametric nonuniformity intensity normalization was applied on volumetric T1-weighted images [24, 25] using FreeSurfer. Then using FSL [26], the following tools were applied sequentially: brain extraction by the BET [27] tool to clear noncerebral voxels; segmentation by the FAST [28] tool into grey matter (GM), white matter (WM), and cerebrospinal fluid (CSF); segmentation by the FIRST tool of left and right hippocampi [29]. Left and right precuneus (PC) and posterior cingulate cortex (PCC) were obtained by an inversion of the nonlinear transformation between the volumetric images and the MNI152 template, on which the AAL template was superimposed. 

Diffusion-weighted images were corrected for motion and eddy current distortion by the FDT tool. After brain extraction, diffusion tensor was reconstructed using an iterative least square algorithm (Marquardt-Levenberg nonlinear fit) to calculate mean diffusivity (MD) and fractional anisotropy (FA) maps. 

A probabilistic tractography of the tract connecting the hippocampus with PC/PCC (Hipp-PC/PCC) was performed on each hemisphere by the FDT tool [30, 31]; first, volumetric T1-weighted image, ROIs of hippocampi, and PC/PCC were coregistered onto diffusion-weighted images, then coregistered hippocampi were used as seed ROI, and coregistered PC/PCC were used as target ROI. Reconstructed tracts were thresholded at 30.

B0 images were normalized onto the EPI template in stereotaxic MNI152 space for each subject. Normalization transformation was applied to all reconstructed tracts. Normalized nonthresholded tracts were binarized, averaged for the three different groups (CTRL, MCI, and AD patients), and then smoothed with a 5 mm Gaussian kernel. 

A voxelwise statistical analysis was performed using the general linear model framework implemented in SPM8 [32]. The resulting t-statistic maps, after family-wise error (FWE) correction for multiple comparisons, were thresholded at *P* < .05. 

The FA and MD maps were co-registered (with a full affine transformation, FLIRT tool [33]) on volumetric brain-extracted images, then this affine transformation was applied to reconstructed thresholded tracts. 

Eventually, average FA and MD were calculated for brain tissue (BT), white matter (WM), grey matter (GM), hippocampi, PC/PCC, and tracts connecting hippocampi with PC/PCC. Absolute volume (mm^3^) and relative volume, expressed as ratio between absolute volume (mm^3^) and intracranial volume (mm^3^), were calculated for BT, WM, GM, hippocampi, PC/PCC, and tracts connecting hippocampi with PC/PCC.

### 2.4. Statistical Analysis

Statistical analysis was performed using SPSS. Average FA values, average MD, values and volumetric values were statistically compared using a Student *t*-test for nonpaired (independent) data between AD and CTRL, between MCI and CTRL, and between MCI and AD. The significant level was set at *P* ≤ .05 for each test. Finally, a Pearson's correlation analysis was performed between MRI data and cognitive scores (MMSE and verbal memory).

## 3. Results

### 3.1. Volumetry Analysis

**Figure 2:**
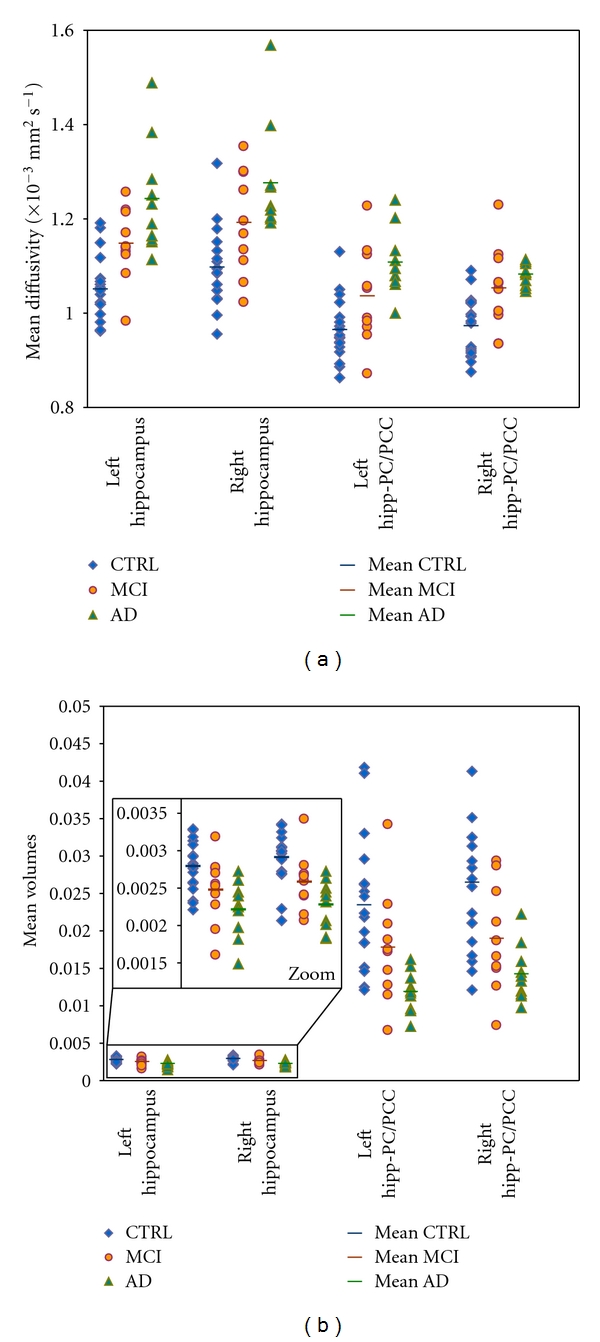
Individual data plotting of MD and relative volumes in hippocampi and correlated structures, in order to estimate the ability of the techniques to discriminate AD, MCI, and healthy controls. (a) Plot of MD values for each subject in left and right hippocampus and in left and right tracts connecting hippocampus to PC/PCC for healthy controls (blue), MCI (orange), and AD (green) subjects. Average MD value is represented by a different color line for each group. (b) Plot of relative volumes values for each subject in left and right hippocampus and in left and right tracts connecting hippocampus to PC/PCC for healthy controls (blue), MCI (orange), and AD (green) subjects. Average relative volume value is represented by a different color line for each group.

Average group values, with respective standard deviations, were reported in Table 2 for relative volumes of each investigated structure. In AD compared to CTRL, volumes of total BT, total GM, and left PC/PCC were significantly decreased (*P* < .05), but even more significantly (*P* = .001 or less) at the level of both hippocampi and tracts connecting hippocampi with PC/PCC. In MCI compared to CTRL, volumes of both hippocampi and tract connecting right hippocampus with PC/PCC were significantly decreased (*P* < .05) compared to CTRL. In AD compared to MCI, volumes of left PC/PCC and tract connecting left hippocampus with PC/PCC were significantly decreased (*P* < .05) (Figure 2(b)).

### 3.2. DTI Analysis

Average group values, with respective standard deviations, were reported in Table 3 for MD of each investigated structure. In AD compared to CTRL, MD of total BT, total WM, and right PC/PCC were significantly increased (*P* < .05), but more significantly increased (*P* < .001) were MD of total GM, left PC/PCC, both hippocampi and both tracts connecting hippocampi with PC/PCC. In MCI compared to CTRL, MD of both hippocampi and both tracts connecting hippocampi with PC/PCC were significantly increased (*P* < .05). In AD compared to MCI, MD of total BT, total GM, and left hippocampus were significantly increased (*P* < .05) (Figure 2(a)).

Average group values, with respective standard deviations, were reported in Table 4 for FA of each investigated structure. In AD compared to CTRL, FA of right hippocampus and tract connecting right hippocampus with PC/PCC were significantly decreased (*P* < .05), but more significantly decreased (*P* < .001 or less) were FA of left hippocampus and tract connecting left hippocampus with PC/PCC. In MCI compared to CTRL, FA of left hippocampus was the only significantly decreased (*P* < .05) compared to CTRL. In AD compared to MCI, FA of tract connecting left hippocampus with PC/PCC was the only significantly decreased (*P* < .05). 

**Figure 3:**
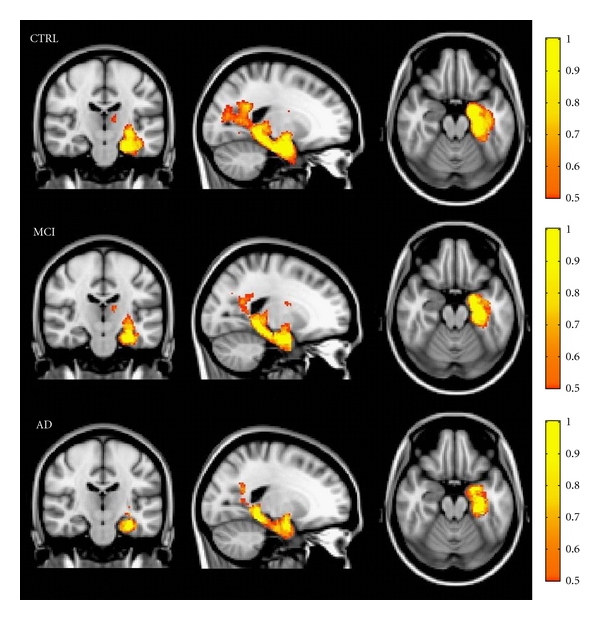
Variability maps for the tract connecting hippocampus with PC/PCC (on MNI152 T1-weighted template) in each subjects group. The color intensity indicates the percentage of subjects in whom the tract passes through each voxel. A yellow color scale indicates the higher degree of overlap among subjects; a red-orange color scale indicates that tract is present only in half of the subjects.

**Figure 4:**
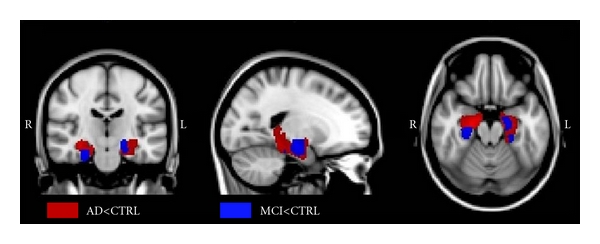
Nonparametric statistical results of voxelwise analysis in SPM8, on probability distribution of the tract connecting hippocampus with PC/PCC in MCI patients, AD patients, and healthy controls (*P* < .05, FWE correction). The red regions represent the areas in which tract connecting hippocampus with PC/PCC is significantly degenerated in AD patients compared to CTRL. The blue regions represent the areas in which this tract is significantly degenerated in MCI patients compared to CTRL.

Results of group analysis for tract connecting each hippocampus with homologous PC/PCC are reported in Figures 3 and 4.  Figure 3 shows reconstructed tracts in CTRL, MCI, and AD patients. Variability maps for these tracts show progressive decrease of volume and consistency from CTRL to AD patients. 

In Figure 4 are shown the results of voxelwise analysis on probability distribution of the tract connecting each hippocampus with homologous PC/PCC in MCI and AD patients compared with CTRL (*P* < .05, FWE correction). In AD (red blobs) patients, probability of connection between hippocampus and PC/PCC was decreased in the whole parahippocampal WM, symmetrically in the two hemispheres. In MCI (blue blobs), probability of connection between hippocampus and PC/PCC was decreased in restricted areas of anterior parahippocampal WM, asymmetrically in the two hemispheres.

### 3.3. Correlation between Volumetric and Diffusion MR Indexes (Table 5)

Volume and MD of total BT and total GM were correlated in CTRL and in MCI, not in AD.

Volume and MD, of hippocampi and PC/PCC were correlated in MCI, not in CTRL, and in AD only of the left hippocampus and the right PC/PCC.

Volume, MD and FA of hipp-PC/PCC tracts were correlated in CTRL and in MCI, but in AD, only volume and MD of the left tract.

### 3.4. Correlation with Cognitive Scores (Table 6)

In AD, volumes of total BT, volumes of total WM, MD of left hippocampus, and MD of left tract connecting hippocampus with PC/PCC correlate significantly (*P* < .05) with verbal memory scores. MD of left tract connecting hippocampus with PC/PCC was the only parameter correlating also with MMSE score. In MCI, FA of total WM was the only parameter correlating significantly (*P* < .05) with verbal memory. No correlation was found between volumetric or DTI parameters and cognitive scores in CTRL.

## 4. Discussion

The aim of the study was to identify sensitive in vivo biomarkers of Alzheimer's disease, to be employed in clinical setting in single patients and to predict the risk for conversion from MCI to AD. The DTI indexes of microstructural damage showed to be at least as sensitive as volumetric indexes of structural damage to identify mesiotemporal damage in differentiating AD and MCI patients from CTRL. The poor correlation between volume and DTI indexes in AD group suggests that macrostructural and microstructural damage may occur in different times in the course of the disease thus may be differently sensitive.

In AD, and to less extent in MCI, atrophy and MD increases were found not only in both hippocampi, but also very significant in white matter tracts connecting hippocampi with precuneus/posterior cingulate cortex. To our knowledge, no study assessed simultaneously atrophy and diffusivity in both hippocampus and parahippocampal-posterior cingulum tract.

We observed also a significant decrease of FA in the hipp-PC/PCC tracts in AD the left tract significantly more than in MCI. The volume of this left hipp-PC/PCC tract, and the volume of left PC/PCC were also found more significantly decreased in AD than in MCI. These results could be explained by a degenerative damage in hipp-PC/PCC tracts, secondary to hippocampal damage. This degenerative damage in hipp-PC/PCC tracts, in agreement with the Villain model, could drive the PC/PCC dysconnection, thus causing in these cortical areas first hypoperfusion in MCI (as found commonly by PET studies, included Villain et al.) and later MD increase and atrophy in AD (as found in the present study, mainly in the left hemisphere). The portion of cingulate tract identified by Villain is included in the tract connecting hippocampi with precuneus/posterior cingulate cortex in our study.

These significant DTI differences between MCI and CTRL groups are not consistently found in literature. Among several DTI studies in MCI, hippocampal MD increase in MCI was found only by few authors [6, 7, 10]. Some studies also found that hippocampal MD detects MCI converters better than hippocampal atrophy [8, 10]. Compared to Muller and Scola, our approach uses a more precise segmentation, specifically of the whole hippocampus as performed in Ray, 2006. While Ray, 2006, used a manual segmentation, our approach with automatic segmentation, still capable to detect early abnormalities in hippocampus of MCI patients, may be more feasible in a clinical setting. Among DTI studies in MCI focusing on white matter tracts, an MD increase in parahippocampal-posterior cingulum was found by few recent studies [34, 35].

It is not known whether damage of parahippocampal-posterior cingulum is the crucial abnormality in MCI converting to AD or not. In the present study, although MD of total BT and total GM differentiated AD from MCI, other significant differences between AD and MCI were found in MD of left hippocampus, FA and volume of left hipp-PC/PCC tract, and volume of left PC/PCC. Therefore, the hippocampus-PC/PCC circuit seems to be specifically involved in AD. While a study using a voxelwise approach [36] failed to find significant MD increase in any white matter region in MCI, the main white matter region with MD increase in early AD was the parahippocampal tract. Another study of the same group, using an ROI-based approach, found that the occipital white matter was found to be the region differentiating better MCI converters from MCI non converters [10]. In that study, however, posterior cingulum and parahippocampal tract were not investigated.

About clinical relevance of volumetric and DTI abnormalities, we looked at correlations with cognitive variables, verbal memory, and MMSE scores. Verbal memory impairment is the earlier crucial deficit in MCI and AD. In this study, in AD the verbal memory score correlated with indexes of global atrophy (total BT, total WM) and at mesiotemporal level only with MD increase, not with atrophy, of left hippocampus and tract connecting left hippocampus with PC/PCC. The clinical relevance of MD increases is also supported by the finding that MD of the tract connecting left hippocampus with PC/PCC was the only variable correlating also with scale of global cognitive status, as expressed by MMSE. In this study, in MCI the verbal memory score correlated only with FA of total WM. Because all MCI patients in this cohort were amnestic MCI, verbal memory was affected in them all, but only few are likely to develop AD in the next years. Therefore, in MCI the correlation of verbal memory score with FA of total WM may reflect a pathological mechanism not specifically related to the Alzheimer's pathology, for instance, to a generic WM damage due to a subtle cerebrovascular disease.

## 5. Conclusions

Both MR volumetric measure of macrostructural damage and DTI measure of microstructural damage, both in grey and white matter, are candidates to be sensitive in vivo biomarkers of mesiotemporal damage predicting conversion from MCI to AD.

DTI abnormalities, especially MD increase, seem to be more clinically relevant than atrophy. 

Only the clinical followup of MCI patients will show which is the most sensitive parameter to be employed to predict the conversion rate on single patients in a clinical setting.

## Figures and Tables

**Table 1:** Demographic and neuropsychological features of CTRL, MCI, and AD patients.

					Student's *t*-test		
Characteristics	CTRL (*N* = 18)	MCI (*N* = 10)	AD (*N* = 10)	*P value	CTRL versus MCI	CTRL versus AD	MCI versus AD
Gender female (*n*, %)	13, 0.72	5, 0.50	8, 0.80	.325	—	—	—
Age (years)	69.1 ± 5.5	70.8 ± 5.9	72.1 ± 4.8	.273	—	—	—
Verbal memory	11.5 ± 2.2	7.3 ± 3.9	4.7 ± 3.2	<.001	0.001	<0.001	n.s.
MMSE	28.8 ± 1.1	25.5 ± 2.3	22.1 ± 2.7	<.001	<0.001	<0.001	0.007

* Statistical analysis: Kruskal-Wallis nonparametric comparison; *P* < .05 is considered significant.

Values are expressed in mean ± SD. CTRL: healthy controls; MCI: mild cognitive impairment; AD: Alzheimer's disease; MMSE: mini-mental state examination.

**Table 2:** Relative volumes in CTRL, MCI, and AD patients.

	CTRL (*N* = 18)		MCI (*N* = 10)		AD (*N* = 10)		Student *t*-test*		
	Mean	(SD)	Mean	(SD)	Mean	(SD)	CTRL versus MCI	CTRL versus AD	MCI versus AD
Brain tissue	0.7635	(0.0188)	0.7550	(0.0250)	0.6733	(0.1791)	n.s.	0.041	n.s.
White matter	0.3951	(0.0194)	0.3841	(0.0274)	0.3385	(0.0881)	n.s.	n.s.	n.s.
Grey matter	0.3684	(0.0151)	0.3709	(0.0131)	0.3349	(0.0927)	n.s.	0.013	n.s.
Left PC/PCC	0.0141	(0.0009)	0.0145	(0.0016)	0.0132	(0.0012)	n.s.	0.033	0.046
Right PC/PCC	0.0128	(0.0009)	0.0126	(0.0015)	0.0120	(0.0011)	n.s.	n.s.	n.s.
Left hippocampus	0.0028	(0.0003)	0.0025	(0.0004)	0.0022	(0.0004)	0.045	<0.001	n.s.
Right hippocampus	0.0029	(0.0003)	0.0026	(0.0004)	0.0023	(0.0003)	0.029	<0.001	n.s.
Left hipp-PC/PCC	0.0234	(0.0087)	0.0178	(0.0075)	0.0119	(0.0027)	n.s.	<0.001	0.031
Right hipp-PC/PCC	0.0265	(0.0099)	0.0190	(0.0071)	0.0143	(0.0038)	0.047	0.001	n.s.

* Statistical analysis: Student *t*-test for independent sample; *P* < .05 is considered significant.

Values are expressed in mean (SD). CTRL: healthy controls; MCI: mild cognitive impairment; AD: Alzheimer's disease; n.s.: not significant; Hipp-PC/PCC: hippocampus to PC/PCC tract.

**Table 3:** Mean Diffusivity (×10^−3^ mm^2^ s^−1^) in CTRL, MCI, and AD patients.

	CTRL (*N* = 18)		MCI (*N* = 10)		AD (*N* = 10)		Student *t*-test*		
	Mean	(SD)	Mean	(SD)	Mean	(SD)	CTRL versus MCI	CTRL versus AD	MCI versus AD
Brain tissue	0.949	(0.038)	0.967	(0.031)	0.996	(0.026)	n.s.	0.002	0.035
White matter	0.809	(0.030)	0.825	(0.023)	0.833	(0.018)	n.s.	0.025	n.s.
Grey matter	0.979	(0.042)	1.006	(0.037)	1.053	(0.036)	n.s.	<0.001	0.011
Left PC/PCC	1.071	(0.045)	1.097	(0.110)	1.170	(0.078)	n.s.	<0.001	n.s.
Right PC/PCC	1.036	(0.045)	1.077	(0.086)	1.128	(0.096)	n.s.	0.002	n.s.
Left hippocampus	1.051	(0.071)	1.147	(0.078)	1.242	(0.117)	0.003	<0.001	0.047
Right hippocampus	1.097	(0.083)	1.191	(0.110)	1.275	(0.120)	0.016	<0.001	n.s.
Left hipp-PC/PCC	0.965	(0.065)	1.036	(0.104)	1.107	(0.070)	0.034	<0.001	n.s.
Right hipp-PC/PCC	0.972	(0.064)	1.052	(0.091)	1.081	(0.024)	0.011	<0.001	n.s.

* Statistical analysis: Student *t*-test for independent sample; *P* < .05 is considered significant.

Values are expressed in mean (SD). CTRL: healthy controls; MCI: mild cognitive impairment; AD: Alzheimer's disease; n.s.: not significant; Hipp-PC/PCC: hippocampus to PC/PCC tract.

**Table 4:** Fractional anisotropy in CTRL, MCI, and AD patients.

	CTRL (*N* = 18)		MCI (*N* = 10)		AD (*N* = 10)		Student *t*-test*		
	Mean	(SD)	Mean	(SD)	Mean	(SD)	CTRL versus MCI	CTRL versus AD	MCI versus AD
Brain tissue	0.207	(0.009)	0.205	(0.008)	0.205	(0.007)	n.s.	n.s.	n.s.
White matter	0.308	(0.017)	0.301	(0.013)	0.305	(0.011)	n.s.	n.s.	n.s.
Grey matter	0.143	(0.007)	0.142	(0.007)	0.139	(0.006)	n.s.	n.s.	n.s.
Left PC/PCC	0.185	(0.017)	0.175	(0.028)	0.174	(0.029)	n.s.	n.s.	n.s.
Right PC/PCC	0.204	(0.021)	0.189	(0.032)	0.190	(0.032)	n.s.	n.s.	n.s.
Left hippocampus	0.134	(0.011)	0.124	(0.012)	0.116	(0.015)	0.032	0.001	n.s.
Right hippocampus	0.133	(0.014)	0.128	(0.012)	0.120	(0.008)	n.s.	0.019	n.s.
Left hipp-PC/PCC	0.215	(0.022)	0.210	(0.025)	0.182	(0.024)	n.s.	0.001	0.019
Right hipp-PC/PCC	0.222	(0.028)	0.205	(0.027)	0.194	(0.019)	n.s.	0.009	n.s.

* Statistical analysis: Student *t*-test for independent sample; *P* < .05 is considered significant.

Values are expressed in mean (SD). CTRL: healthy controls; MCI: mild cognitive impairment; AD: Alzheimer's disease; n.s.: not significant; Hipp-PC/PCC: hippocampus to PC/PCC tract.

**Table 5:** Pearson's correlation between volumetric and diffusion MR indexes.

Volumetric variable	Diffusion variable	CTRL (*N* = 18)	MCI (*N* = 10)	AD (*N* = 10)
Volume brain tissue	MD brain tissue	0.001	<0.001	n.s.
Volume grey matter	MD grey matter	0.001	0.008	n.s.
Volume left PC/PCC	MD left PC/PCC	n.s.	0.014	n.s.
Volume right PC/PCC	MD right PC/PCC	n.s.	0.004	0.004
Volume left hippocampus	MD left hippocampus	n.s.	0.001	0.002
Volume right hippocampus	MD right hippocampus	n.s.	0.003	n.s.
Volume left hipp-PC/PCC	FA right hippocampus	0.036	n.s.	n.s.
	MD left hipp-PC/PCC	<0.001	<0.001	0.002
	FA left hipp-PC/PCC	<0.001	0.015	n.s.
Volume right hipp-PC/PCC	MD right hipp-PC/PCC	<0.001	<0.001	n.s.
	FA right hipp-PC/PCC	0.004	0.002	n.s.

Statistical analysis: 2-tailed Pearson's correlation; *P* < .05 is considered significant.

Only significant correlations were reported. MCI: mild cognitive impairment; AD: Alzheimer's Disease; n.s.: not significant; Hipp-PC/PCC: hippocampus to PC/PCC tract.

**Table 6:** Pearson's Correlation between MR indexes and cognitive scores.

	MCI (*N* = 10)		AD (*N* = 10)	
	Verbal memory	MMSE	Verbal memory	MMSE
Relative BT volume	n.s.	n.s.	0.031	n.s.
Relative WM volume	n.s.	n.s.	0.019	n.s.
MeanFA BT	0.045	n.s.	n.s.	n.s.
MeanMD left hippocampus	n.s.	n.s.	0.048	n.s.
MeanMD left hipp-PC/PCC	n.s.	n.s.	0.029	0.037

Statistical analysis: 2-tailed Pearson's correlation; *P* < .05 is considered significant.

Only significant correlations were reported. MCI: mild cognitive impairment; AD: Alzheimer's disease; n.s.: not significant; MMSE: mini-mental state examination; Hipp-PC/PCC: hippocampus to PC/PCC tract.
